# The Spectrum of Non-Parkinsonian Tremor: A Registry at a Tertiary Care Teaching Institute

**DOI:** 10.5334/tohm.828

**Published:** 2023-12-21

**Authors:** Sanjay Pandey, Shreya Dinesh, Chandra Shekhar Rawat, B. K. Thelma

**Affiliations:** 1 Department of Neurology, Govind Ballabh Pant Institute of Postgraduate medical education and Research, New Delhi, India; 2 Department of Neurology and Stroke Medicine, Amrita Hospital, Mata Amritanandamayi Marg Sector 88, Faridabad, Delhi National Capital Region, India; 3 Department of Microbiology and Bioinformatics, Aravind Medical Research Foundation, Madurai, Tamil Nadu, India; 4 Department of Genetics, University of Delhi South Campus, New Delhi, India

**Keywords:** Dystonic tremor, Essential tremor, Essential tremor plus

## Abstract

**Background:**

Tremors other than those associated with Parkinson’s disease (non-parkinsonian tremor) are commonly observed in clinical settings. However, their frequency and clinical characteristics have rarely been reported.

**Objectives:**

To classify non-parkinsonian tremors based on the consensus statement on the classification of tremors, from the task force of the International Parkinson and Movement Disorder Society published in 2018.

**Methods:**

A prospective registry at a tertiary care teaching institute.

**Results:**

A total of 475 patients with non-parkinsonian tremors were recruited for the study. 67.57% (n = 321) of our patients were male and a family history of tremor was present in 20.84% (n = 99) of patients. Dystonic tremor (DT) was the most common non-parkinsonian tremor (33.26%). 27.78% of patients fulfilled the new classification criteria for essential tremor, with 13.47% classified as pure ET (ET) and 14.31% exhibiting neurological soft signs, leading to the classification of ET plus (ETP). Patients with ETP had more family history (57.35%) [vs DT (26.48%, p = 0.00004) and ET (10.93%, p = 0.00003], longer duration of disease [mean ± standard deviation (SD) = 9.53 ± 8.64 years] [vs DT (5.60 ± 5.93, p = 0.0003) and ET (6.38 ± 5.97, p = 0.01) years], and more severe tremor as measured by the essential tremor rating assessment scale total score [mean ± SD = 27.42 ± 11.70] [vs DT (23.50 ± 8.62, p = 0.007) and ET (22.12 ± 8.19, p = 0.007)] compared with patients with DT and ET.

**Conclusions:**

DT was the most common cause of non-parkinsonian tremor in our registry followed by essential tremor syndrome. ETP was more common than ET.

Tremors other than those associated with Parkinson’s disease (PD) are frequently encountered in clinical practice [1]. Essential tremor (ET) has been considered to be the most common cause of non-parkinsonian tremor [2]. However, the diagnosis of ET remains a clinical one, and diagnostic errors are quite common, with frequent misclassification concerning other movement disorders, especially dystonia, enhanced physiological tremor, and PD [3,4]. Also, ET is more likely to have an alternate diagnosis. Sometimes, the diagnosis is overapplied and used as a “waste-basket” for a variety of tremor disorders, including dystonic tremor (DT) [5]. In recent years there have been many advancements in ET research including a proposed definition and new terminology ‘Essential tremor plus (ETP)’ [6,7]. The consensus criteria propose that ET should be defined as an isolated tremor syndrome with a 3-year duration. ETP is defined as patients with the characteristics of ET and additional neurological signs of uncertain significance (soft signs) such as questionable dystonia, impaired tandem gait, and memory impairment [6]. However, ETP is a controversial concept as it has not been defined based on a definite underlying pathology [8,9,10,11].

This prospective study was performed to assess the spectrum of non-parkinsonian tremors and describe their demographic and clinical features. To, the best of our knowledge this is the first prospective study of patients with non-parkinsonian tremor following the new consensus classification.

## Methods

A total of 475 patients with ‘non-parkinsonian Tremor syndromes’ attended our tertiary care movement disorder center at Govind Ballabh Pant Institute of Postgraduate Medical Education and Research, New Delhi, India between January 2019 to July 2021. Classification of tremor was performed using the 2018 consensus criteria [6]. The study was approved by our institutional ethics committee and informed written consent for participation in the study was taken from all patients. Clinical and demographic data were recorded using a structured proforma. Response to alcohol was recorded from all patients. Family history of tremors was obtained from first-degree relatives and questions were asked about the presence or absence of tremors in them. All patients were examined by two neurologists (SP, CSR). Neurological examination was videotaped for 3–5 minutes to record the tremor and other soft signs. Upper limb tremor was assessed in the true rest position, with hands pronated and resting on their lap; postural tremor was assessed during the forwarding position of both hands for 5 seconds and in the wing-beating position of both upper limbs for 20 seconds. Intention tremor was assessed by a finger-nose-finger manoeuvre repeated 3 times. Lower limb tremor was assessed at rest, during posture by raising each limb horizontally, parallel to the ground, for 5 seconds each, and then by performing a standard heel-to-shin manoeuvre with each leg 3 times. Cranial tremors were noted in the head, face, and voice. The head tremor was assessed at rest, by rotating the head fully to the left and right and then was observed for 10 seconds in mid-position. The patient was then instructed to gaze fully to the left and then to the right with the head in mid-position. Voice tremor was assessed during sustained phonation while reading prepared paragraphs and during speech. The essential tremor rating assessment scale (TETRAS) was used to assess the severity of the tremor [12]. Patients with bradykinesia were excluded. A tremor in a body part affected by dystonia was labelled as dystonic tremor (DT). Dystonia was labelled as questionable if there was discordance between the two examiners (S.P., CSR) regarding its presence. If dystonia and tremor were found in different body parts, this was called tremor associated with dystonia (TAWD). Tandem gait impairment was noted by asking each subject to walk tandem (place one foot in front of the other touching toe to heel) and the number of missteps during 10 steps was counted. Tandem walk abnormalities were based on the observation of two or more missteps [13]. Cognitive function assessment was done using the ‘Montreal Cognitive Assessment’ (MOCA) test. A score below 26 with no functional impairment indicated mild cognitive impairment [14].

The data were entered onto a Microsoft Office Excel sheet and statistical analysis was done using the IBM-SPSS statistics 27 version. Values were expressed as means ± standard deviations [SD] and as percentages and ranges. The mean between the two groups was compared using the *t*-test, frequencies between the various groups were compared using the χ^2^ test, and *p* values ≤ 0.05 were considered statistically significant. The multiple comparisons were corrected using the Benjamini-Hochberg approach [15].

## Results: (Tables 1 and 2, Figure 1)

**Table 1:** Number of patients with non-parkinsonian tremor syndromes.


NON-PARKINSONIAN TREMOR SYNDROME	NUMBER OF PATIENTS (N = 475)	

**Dystonic tremor**	158 (33.26%) Body distribution of dystonia: **Focal: 86 (54.43%)** Cervical: 60 Focal hand dystonia: 22 Leg: 4 **Segmental: 60 (37.97%)** Craniocervical: 39, Craniocervicobrachial: 21 **Multifocal: 3 (1.89%)** **Generalized: 9 (5.69%)**	

**Essential tremor plus**	68 (14.31%) **Soft signs:** ***Questionable dystonia: 64*** Bilateral upper limb + Neck: 32 Only upper limb: 19 Only neck: 13 [Upper limb: unilateral (n = 32); bilateral (n = 19)] [Neck: 32+13 = 45; (Torticaput = 18, laterocollis = 12, laterocaput = 6, retrocollis = 6, anterocollis = 2, lateral shift = 1)] ***Mild cognitive impairment: 12*** ***Impaired tandem gait: 13*** ***Rest tremor: 4***	

**Essential tremor**	64 (13.47%)	

**Functional tremor**	26 (5.47%)	

**Indeterminate tremor**	20 (4.21%)	

**Isolated Head tremor**	9 (1.89%)	

**Primary writing tremor**	6 (1.26%)	

**Acquired causes of tremor**	124 (26.10%)	

***Details of acquired causes***	Drug induced	80 (64.51%) [Antiepileptics-70; Antipsychotics-5;Alcohol-3, Beta-agonist-inhalers-1, Metronidazole-1]

	Stroke	14 (11.29%)

	CNS infection	10 (8.06%)

	Neuropathy	7 (5.64%)

	Holmes’ tremor	4 (3.25%)

	Cervical myelopathy	3 (2.49%)

	Multiple sclerosis	1(0.08%)

	Hypercalcemia	1(0.08%)

	Mitochondrial disorder	1(0.08%)

	Sub-acute-combined degeneration	1(0.08%)

	Infantile-tremor syndrome	1(0.08%)


**Table 2:** Demographic and clinical features of patients with non-parkinsonian tremor syndromes.


TYPE OF TREMOR	DT	ETP	ET	ET VS ETP (^*^P VALUE)	DT VS ET (P VALUE)	DT VS ETP (P VALUE)	ACQUIRED TREMOR	FT	IDT	IHT	PWT

**Number of patients**	158	68	64	–	–	–	124	26	20	9	6

**Family history present**	45 (28.48%)	39 (57.35%)	7 (10.93%)	**0.00003**	**0.005**	**0.00004**	3 (2.41%)	2 (7.6%)	1 (5%)	1 (11%)	1 (16.66%)

**Gender** **M:F**	98/60	51/17	55/9	0.11	**0.001**	0.07	84:40	10:16	12:8	5:4	6:0

**Response to alcohol**	17 (10.75%)	16 (23.52%)	19 (29.68%)	0.42	**0.003**	**0.01**	9 (7.25%)	0	3 (15%)	2 (22.22%)	1 (16.66%)

**Mean age** ± **SD (range) years**	45.34 ± 16.37 (11–80)	48.32 ± 17.98 (13–80)	47.79 ± 18.75 (16–80)	0.86	0.49	0.49	35.97 ± 16.68 (1.5–73)	34.42 ± 11.30 (12–58)	26.2 ± 11.23 (13–50)	58.77 ± 9.36 (40–73)	39.83 ± 16.63 (22–60)

**Mean duration of disease** ± **SD (range) years**	5.60 ± 5.93 (0.5–40)	9.53 ± 8.64 (3–50)	6.38 ± 5.97 (3–30)	0.01	0.37	**0.0003**	2.99 ± 4.39 (0.02–30)	1.61 ± 1.76 (5 days–7 years)	1.25 ± .58 (0.5–2.5)	8.66 ± 8.14 (0.5–30)	2.16 ± 1.24 (0.5–4 years)

**Mean TETRAS-A** ± **SD (range)**	13.81 ± 6.40 (2–40)	12.15 ± 5.76; (2–27)	12.78 ± 5.93 (2–30)	0.53	0.39	0.18	7.26 ± 8.03 (1–40)	11.21 ± 4.26 (2–28)	2.65 ± 0.85 (1–5)	18.33 ± 9.10 (11–39)	12.0 ± 2.0 (9–15)

**Mean TETRAS-P** ± **SD (range)**	9.68 ± 3.70 (2–27)	15.26 ± 6.49; (3–31.5)	9.34 ± 3.82 (2–27)	**0.0001**	0.94	**0.0001**	8.37 ± 4.97 (1–23)	8.50 ± 3.41 (2–12.5)	4.65 ± 2.26 (1–9)	13.88 ± 5.87 (8–28)	7.5 ± 2.14 (5–11)

**Mean TETRAS-T** ± **SD (range)**	23.50 ± 8.62 (4–67)	27.42 ± 11.70; (6–54.5)	22.12 ± 8.19 (6–49)	**0.007**	0.27	**0.007**	15.56 ± 12.04 (3–63)	19.71 ± 6.75 (9–44)	7.4 ± 2.41 (3–12)	32.22 ± 13.65 (19–58)	19.5 ± 3.20 (17–26)


M: Male; F: Female; SD: Standard deviation; DT: Dystonic tremor; ET: Essential tremor; ETP: Essential tremor plus; TETRAS: The Essential Tremor Rating Assessment Scale; TETRAS-A: TETRAS activities of daily living subscale; TETRAS-P: TETRAS performance subscale; TETRAS-T: TETRAS total score; FT: Functional tremor; IDT: Indeterminate tremor; IHT: Isolated head tremor; PWT: Primary writing tremor; ^*^Bold indicates significant p value.

**Figure 1:**
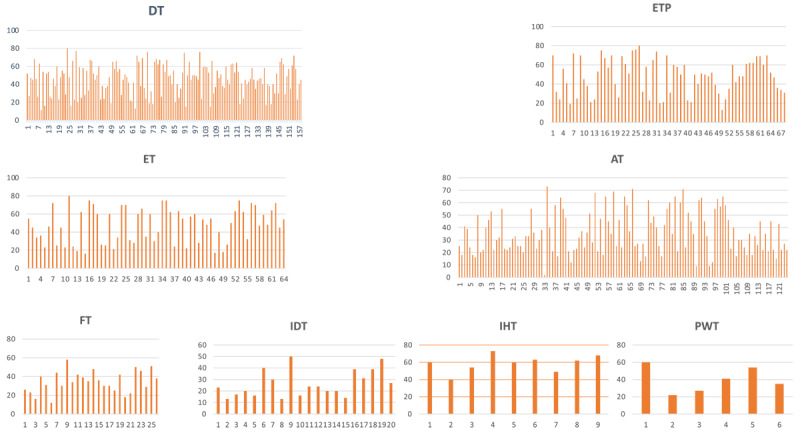
Age of patients with different non-parkinsonian tremor syndromes. x-axis: Number of patients; y-axis: age of the patients. DT: Dystonic tremor; ETP: Essential tremor plus; ET: Essential tremor; AT: Acquired tremor; FT: Functional tremor; IDT: Indeterminate tremor; IHT: Idiopathic head tremor; PWT: Primary writing tremor.

A total of 475 patients with non-parkinsonian tremor were recruited for our study. 67.57% (n = 321) of our patients were male and a family history of tremor was present in 20.84% (n = 99) of patients. DT was the most common type of non-parkinsonian tremor (n = 158; 33.26%) observed. TAWD was present in 43.03% (68/158) of these patients. 27.78% (n = 132) of patients fulfilled the new classification criteria for essential tremor, with 13.47% (n = 64) classified as pure ET (ET) and 14.31% (n = 68) exhibiting neurological soft signs, leading to the classification of ET plus (ETP). Other types of tremors were classified as acquired tremor (n = 124; 26.10%), functional tremor (n = 26; 5.47%) indeterminate tremor (n = 20; 4.21%), isolated head tremor (IHT) (n = 9; 1.89%), and primary writing tremor (n = 6; 1.26%).

## Comparison of patients with DT, ETP, and ET

The majority of our patients with DT (62.02%), ETP (75%), and ET (85.93%) were males. A positive family history was more common in patients with ETP (57.35%), compared to patients with DT (26.48%) and ET (10.93%). The mean ± SD duration of the disease was longer in patients with ETP (9.53 ± 8.64), compared to patients with ET (6.38 ± 5.97) and DT (5.60 ± 5.93). The tremor severity as measured by mean ± SD TETRAS-T (total score) was higher in patients with ETP (27.42 ± 11.70) compared to patients with DT (23.50 ± 8.62) and ET (22.12 ± 8.19).

## Acquired tremor

Acquired tremor (AT) was present in 124 (26.10%) patients and 67.74% (n = 84) of them were males. The mean age ± SD (range) of patients with AT was 35.97 ± 16.68 (1.5–73) years and the mean ± SD (range) duration of symptoms was 2.99 ± 4.39 (0.02–30) years. The most common cause of AT was a drug (64.51%), followed by stroke (11.29%), central nervous system infection (8.06%), peripheral neuropathy (4.64%), Holmes’ tremor (3.25%), and cervical myelopathy (2.49%). Antiepileptic drugs (87.5%) were the most common cause of drug-induced tremor.

## Discussion

We have recruited 475 patients with non-parkinsonian tremor syndrome who were classified based on the recent consensus classification [6]. In our cohort, DT was the most common tremor syndrome (n = 158, 33.26%) followed by ETP (n = 68, 14.31%) and ET (n = 64, 13.47%) combined (n = 132; 27.78%). In the past, a major problem has been that specialists differed in their definition of ET leading to misdiagnosis in a large number of cases [16]. This resulted in previous studies reporting dystonia in approximately 50% of patients with ET [17,18,19]. The new definition has included the terminology ‘Essential tremor plus’ to define ET patients with additional neurological signs of uncertain significance (soft signs) such as questionable dystonia, impaired tandem gait, and memory impairment [6]. Interestingly, we have observed a greater number of patients with ETP compared with ET. Our findings are consistent with many recent studies where existing ET cohorts diagnosed using the 1998 consensus criteria were reclassified and patients were more likely to be classified as ETP than ET [20,21,22]. Also, there is a grey zone between ETP and ET with aging and long duration of disease [21,22,23]. Furthermore, there are issues with inter-examiner variability in terms of validity of abnormal posture (between ET and ETP) and final diagnosis (ET and ET with aging and long duration). In a recent prospective study, we observed that older patients with ETP had more soft signs including questionable dystonia [24]. These findings suggest that ETP is likely to be just a later stage of what has been labelled as ET [25]. So, it is important to determine whether the new neurological soft signs in ETP are pathologically/etiologically related or coincidental [25]. Another problem in a clinical setting is to differentiate ETP from DT [22]. In the absence of a clear definition of questionable dystonia, a diagnosis of ETP or DT remains observer-dependent with a high degree of interrater disagreement [22,26]. Certain clinical signs such as lower limb action tremor, highly asymmetric upper limb tremor, unusual postures (e.g., finger pointing), mirror dystonia, muscular overflow contractions, irregular rhythm and jerkiness, and isolated head or voice tremor are incompatible with ET and more likely to suggest DT [27,28]. Additionally, certain electrophysiological techniques such as surface electromyography with an accelerometer, blink reflex recovery curve, and somatosensory temporal discrimination threshold may be useful in differentiating ET and DT [19,29,30]. However, they are not easily available and not validated. Neuroimaging and genetics have also been used as possible biomarkers for ET and DT [31,32].

Our study findings should be interpreted with some inherent limitations. First, our movement disorder center is a tertiary care referral center leading to an inherent bias regarding the patients referred here. Second, the tremor was assessed by multiple raters with no inter-rater reliability data. Third, the male dominance observed in our study may be due to social rather than biological factors considering that there is a gender difference in health expenditures and treatment-seeking behaviour among patients [33]. Fourth, we did not perform electrophysiological studies on all patients. Fifth, the exact mechanism of tremor in some of the patients with acquired tremor secondary to cervical myelopathy may be unclear [34].

Despite these limitations, our study has provided detailed demographic and clinical characteristics of different types of non-parkinsonian tremors. DT was the most common type of non-parkinsonian tremor. Also, the number of patients with ETP was more than patients with ET, which is consistent with the many retrospective studies published after the new consensus classification. Patients with ETP had more family history, longer duration of disease, and more severe symptoms. Furthermore, a significantly higher proportion of family history in patients with ETP may indicate major genetic determinants in disease etiology warranting continued disease gene discovery efforts. Considering the single-center study our findings need to be validated in a multicentric study recruiting a large number of patients.
